# ENHANCING CARE AND TEAM PROCESSES THROUGH NURSE PRACTITIONER-LED HUDDLES IN LONG-TERM CARE HOMES

**DOI:** 10.1093/geroni/igae098.1952

**Published:** 2024-12-31

**Authors:** Shirin vellani, Katherine McGilton, Alexandra Krassikova, Margaret Keatings, Souraya Sidani

**Affiliations:** Toronto Rehabilitation Institute-UHN - Toronto, ON, toronto, Ontario, Canada; Toronto Rehabilitation Institute-UHN - Toronto, ON, toronto, Ontario, Canada; KITE Research Institute, Toronto, Ontario, Canada; Toronto Rehabilitation Institute, Toronto, Ontario, Canada; Toronto Metropolitan University, Toronto, Ontario, Canada

## Abstract

Long-term care (LTC) homes faced unprecedented challenges during the COVID-19 pandemic, impacting both staff and residents. Huddles led by a skilled facilitator have the potential to enhance care and team dynamics positively. Nurse practitioners (NPs) are highly trained clinician and leader with a proven history of contributing to favorable outcomes for residents, staff, and the health system within LTC homes. The objectives of this mixed-methods study included: 1) assess the implementation of NP-led huddles; 2) compare outcomes of moral distress and perceived support between staff; and 3) examine changes in resident outcomes. Over a four-month period, huddles were conducted on two units of a privately-owned, not-for-profit LTC home in Ontario, Canada. Post-implementation outcomes were compared between staff who attended at least one huddle (intervention group, n=20) and those who did not (control group, n=22). Anonymized resident-level data from RAI MDS 2.0 were utilized to evaluate resident outcomes. Bayesian analysis was employed to compare outcomes across different staff categories and to summarize changes in RAI measures before and after the intervention. Forty-eight huddles, primarily focusing on resident care (46%) and staff well-being (34%), were conducted by the NP. Direct care staff who attended huddles reported lower levels of moral distress and increased support from the NP. One of the intervention units showed statistical evidence of reduced medical complexity among residents over time. In conclusion, NP-led huddles have the potential to positively impact both staff and resident outcomes and integration of NPs into LTC can facilitate implementation of evidence-informed practices in LTC.

